# Cost-effectively dissecting the genetic architecture of complex wool traits in rabbits by low-coverage sequencing

**DOI:** 10.1186/s12711-022-00766-y

**Published:** 2022-11-18

**Authors:** Dan Wang, Kerui Xie, Yanyan Wang, Jiaqing Hu, Wenqiang Li, Aiguo Yang, Qin Zhang, Chao Ning, Xinzhong Fan

**Affiliations:** College of Animal Science and Veterinary Medicine, Shandong Agricultural University, Tai’an, China

## Abstract

**Background:**

Rabbit wool traits are important in fiber production and for model organism research on hair growth, but their genetic architecture remains obscure. In this study, we focused on wool characteristics in Angora rabbits, a breed well-known for the quality of its wool. Considering the cost to generate population-scale sequence data and the biased detection of variants using chip data, developing an effective genotyping strategy using low-coverage whole-genome sequencing (LCS) data is necessary to conduct genetic analyses.

**Results:**

Different genotype imputation strategies (BaseVar + STITCH, Bcftools + Beagle4, and GATK + Beagle5), sequencing coverages (0.1X, 0.5X, 1.0X, 1.5X, and 2.0X), and sample sizes (100, 200, 300, 400, 500, and 600) were compared. Our results showed that using BaseVar + STITCH at a sequencing depth of 1.0X with a sample size larger than 300 resulted in the highest genotyping accuracy, with a genotype concordance higher than 98.8% and genotype accuracy higher than 0.97. We performed multivariate genome-wide association studies (GWAS), followed by conditional GWAS and estimation of the confidence intervals of quantitative trait loci (QTL) to investigate the genetic architecture of wool traits. Six QTL were detected, which explained 0.4 to 7.5% of the phenotypic variation. Gene-level mapping identified the *fibroblast growth factor 10* (*FGF10*) gene as associated with fiber growth and diameter, which agrees with previous results from functional data analyses on the FGF gene family in other species, and is relevant for wool rabbit breeding.

**Conclusions:**

We suggest that LCS followed by imputation can be a cost-effective alternative to array and high-depth sequencing for assessing common variants. GWAS combined with LCS can identify new QTL and candidate genes that are associated with quantitative traits. This study provides a cost-effective and powerful method for investigating the genetic architecture of complex traits, which will be useful for genomic breeding applications.

**Supplementary Information:**

The online version contains supplementary material available at 10.1186/s12711-022-00766-y.

## Background

Genome-wide association studies (GWAS) have delivered new insights into the biology and genetic architecture of complex traits. In the past decades, GWAS has accelerated the rate of gene discovery to an unprecedented scale, identifying many replicated genetic variants associated with complex diseases and quantitative traits in livestock, plants, humans, and model organisms [1–4]. Phenotypic variation in complex traits is often caused by the cumulative effect of numerous common variants, i.e., such traits are polygenic, and thus high-density GWAS can provide novel insights into their genomic architecture [5]. Traditional high-density GWAS requires two distinct genetic testing technologies: high-coverage sequencing of whole genomes and a genome-wide genotyping array followed by imputation. Considering the cost to generate population-scale sequence data and a lack of inexpensive high-density chips, low-coverage whole-genome sequencing (LCS) followed by imputation is a much more affordable alternative for assessing common genetic variants and testing the association of millions of variants with phenotype for complex traits [6] and can increase the discovery power of trait-associated and/or causal genetic variants [6, 7]. To date, LCS has been widely used to accurately assess common variants using GWAS. It has been shown that 0.5 to 1X LCS achieved comparable performance to commonly used low-density GWAS arrays [8]. LCS at a depth of 1X was able to detect signals that were missed by standard imputation of single nucleotide polymorphism (SNP) arrays [9]. A more systematic examination of the power of GWAS suggested that 1X LCS allows the identification of up to twice as many associations as standard SNP array imputation [10]. Furthermore, LCS at a depth of ≥ 4X can capture variants with various frequencies more accurately than all commonly used GWAS arrays, at a comparable cost [8].

The approach of LCS followed by imputation exploits the fact that individuals in the same cohort are sufficiently related to share large genome segments [7]. Missing genotypes in LCS data are imputed using local linkage patterns to infer unknown genotypes in target samples from known genotypes. The current available tools for imputation of LCS data include STITCH [11], Beagle [12], GeneImp [13], GLIMPSE [14], and loimpute [15], some of which use a haplotype reference panel. STITCH [11] imputes genotypes based only on sequencing read data, without requiring additional reference panels or array data, and is applicable in settings of extremely low sequencing coverage [16, 17]. The other tools are based on the use of reference panel information, for example, GLIMPSE phases and imputes LCS data using large reference panels [14]. In addition, Beagle was developed for genotype imputation and is tailored to work both with and without reference panels [12]. Since the costs of both library construction and sequencing are continuingly decreasing, LCS has become increasingly attractive to obtain genotyping information for farm animals [17].

Angora rabbits are particularly well known for their wool production. The economic value of Angora wool depends on the texture of the hair, which is mainly characterized by fiber diameter and length. In this study, we generated accurate and dense genotype data on Angora rabbits with a cost-efficient LCS approach and evaluated imputation performance across five levels of sequencing coverage and six levels of sample size, using three imputation strategies (BaseVar + STITCH, Bcftools + Beagle4, and GATK + Beagle5). To decipher the genetic architecture of complex wool traits in Angora rabbits, we performed GWAS of six important economic traits at various time points with high resolution. We also developed a conditional GWAS and estimated the confidence intervals of identified quantitative trait loci (QTL) by the drop (∆) in log-transformed *P* values method in a multivariate linear mixed model, which were used to identify candidate genes.

## Methods

The experimental procedures used in this study were approved by the Animal Care and Use Committee of Shandong Agricultural University.

### Animals and phenotypes

In total, 629 Angora rabbits (298 males and 331 females) from the same batch were used in this study. The rabbits were raised in five houses on one farm under the same conditions, including diet, water and temperature. Since rabbits are artificially inseminated with mixed semen in standard production conditions, no precise pedigree information was available for the studied population. Wool traits were measured at 70, 140 and 210 days of age and included length of fine wool (LFW), diameter of fine wool (DFW), coefficient of variation of diameter of fine wool (CVDFW), length of coarse wool (LCW), and rate of coarse wool (RCW). Wool samples were obtained from the center of the lateral body by shaving with clippers. Body weight (BW) was measured at weaning weight at 35 days and at 70, 140, and 210 days of age.

### Sequencing

Ear samples were collected from each individual. Genomic DNA was isolated using the Qiagen MinElute Kit. Genomic DNA from each sample was used to construct a paired-end library (PE150) with ~ 350-bp inserts. All libraries were sequenced on the DNBSEQ-T7 platform. An average of 3.84X genomic coverage for 627 samples was sequenced, with the read depth ranging from 1.51X to 8.03X. In addition, 15 samples were deep-sequenced at 10X coverage for genotype validation. In total, 7305 gigabases of genomic sequence data were generated.

### Preprocessing of sequence data

Read quality was assessed using the FastQC tool (https://www.bioinformatics.babraham.ac.uk/projects/fastqc/) with a focus on base quality scores, GC content, N content, and sequence duplication levels. Adapters and low-quality bases were removed using Trimmomatic as “java -jar trimmomatic-0.38.jar PE sample_1.fq.gz sample_2.fq.gz 1_paired.fq.gz 1_unpaired.fq.gz 2_paired.fq.gz 2_unpaired.fq.gz ILLUMINACLIP:TruSeq3-PE.fa:2:30:10 SLIDINGWINDOW:5:20 LEADING:5 TRAILING:5 MINLEN:50” [18]. Sample reads were mapped to the rabbit reference sequence GCF_000003625.3 (*Oryctolagus cuniculus*) using the BWA-mem algorithm [19]. All PCR duplicates were removed using Picard tools (https://broadinstitute.github.io/picard/).

### Genotyping using high-depth sequencing

Variant calling was performed using the GATK4 best practices [20]. Base quality score realignment and recalibration were applied to each sample and the haplotypecaller software was used for variant discovery. Average coverage was estimated using Qualimap 2.2.1. To simulate low-pass sequencing, the 15 BAM files were down-sampled to 0.1X, 0.5X, 1.0X, 1.5X and 2.0X coverage using Picard.

### Genotype imputation using low-coverage sequencing

Due to the lack of a reference panel, we used imputation tools that do not require reference information, i.e. STITCH and Beagle, to impute genotypes using the low-coverage sequencing data. Error and bias towards the reference allele occur in SNP calling using low-coverage data because of its limited information and when using standard tools that are designed for high-coverage data, such as SAMtools followed by Bcftools and GATK [21]. BaseVar (https://github.com/ShujiaHuang/basevar) was developed to call variants for large-scale low-pass whole-genome sequencing (WGS) data and was suitable for our study. Useful discussions on the development of the BaseVar to call SNPs are reported in [16]. In addition, several articles (e.g. [22, 23]) have documented the reasons why BaseVar is preferred for low-coverage sequencing data. In this study, BaseVar was mainly applied to identify variant sites, and STITCH was used to impute SNPs. The performance of Bcftools and GATK in SNP calling of LCS data was also tested, along with Beagle to impute SNPs. Hence, three imputation pipelines were compared including: (1) BaseVar + STITCH: SNPs were called using BaseVar and site information was provided to STITCH [11] to impute genotype probabilities and output imputed dosage genotypes and genotypes in VCF format; (2) Bcftools + Beagle4 (genotype likelihoods): SNPs were called using Bcftools [22] and then Beagle v4.1 [23] was conducted to impute genotype probabilities and output imputed dosage genotypes and genotypes in VCF format; and (3) GATK + Beagle5: SNPs were called using GATK [20] and then Beagle v5.1 [24] was conducted to impute genotypes and output imputed genotypes in VCF format. The two versions of Beagle that were used to impute genotypes based on different types of data, i.e. Beagle v4.1 infers genotypes from genotype likelihood input data, whereas Beagle v5.1 uses genotype data and provides significantly faster genotype phasing and similar imputation accuracy [24].

### Assessing imputation accuracy

Genotypes that are directly called from high-coverage sequencing are the de facto standard for validating the imputation of untyped SNPs. Here, imputation accuracy was assessed for the three cost-effective genotype imputation strategies by comparing imputed genotypes to high-coverage genotypes and was measured by two criteria, i.e. genotype concordance (GC) and genotype accuracy (GA), by identifying sites that are shared across the two datasets. Sites were considered shared if their position, reference allele, and alternate allele were identical. Genotype concordance was defined as the proportion of imputed genotypes that were identical to the genotype determined using high-coverage sequencing. For each site, GC was set to 0 if the imputed genotype did not match the true genotype and to 1 if the imputed genotype did match the true genotype. Thus, GC of a site was calculated as the average over all the samples at that site. Genotype accuracy (GA) was defined as the Pearson correlation coefficient between imputed genotypes and genotypes obtained by high-coverage sequencing. Compared to GC, GA was calculated based on imputed genotype probabilities [25]. Furthermore, in order to examine the influence of the number of samples and sequence coverage on imputation, we compared GC and GA for different sample sizes (100, 200, 300, 400, 500 and 600) and sequencing depths (0.1X, 0.5X, 1.0X, 1.5X, 2.0X) by down-sampling.

### Selection of tagging SNPs and annotation

According to Teng et al. [26], when imputation is done with STITCH, it is necessary to complete the remaining missing variants using Beagle. Thus, we also carried out such two-stage imputation, i.e., “Basevar-Stitch-Beagle5”. For convenience, we kept the “BaseVar + STITCH” name in the following section on genetic analyses. The SNPs (directly genotyped and imputed by STITCH) were filtered for an imputation info score > 0.4 using Bcftools, and then for a minor allele frequency (MAF) > 0.05, a genotype missing rate < 0.1, and a Hardy–Weinberg equilibrium (HWE) *p*-value > 1 × 10^–6^ using the PLINK software [27]. The sites, which were missing in 10% of the individuals after STITCH imputation, were then imputed by Beagle v5.1. SNPs were annotated and categorized as polymorphisms in exonic regions, intronic regions, and intergenic regions using ANNOVAR [28] based on the rabbit reference genome, and SNPs in exons were further classified into synonymous or non-synonymous SNPs.

### Population genetics analysis

We performed principal component analyses (PCA) on the population of 629 rabbits using the GCTA software [29]. The first five principal components were extracted and visualized in R. Linkage disequilibrium (LD) decay was measured across whole genomes by calculating r^2^ values with the PopLDdecay software [30]. In order to detect regions of signatures of selection, the composite likelihood ratio (CLR) statistic test was implemented using the software SweeD [31]. Nucleotide diversity (Pi) was calculated using Vcftools [32] simultaneously with a window size set to 50 kb and a step size of 10 kb. Sliding windows along the whole genome with the top 1% of CLR and Pi values were regarded as putative regions with signatures of selection.

Domestication and centuries of selective breeding have changed the genomes of rabbit breeds to adapt to environmental challenges and human needs. In order to explore and further detect signatures of selection, 14 domesticated rabbits were sampled from the population to analyze their genetic diversity and population structure compared to 14 wild rabbits, i.e. their wild progenitor. A maximum-likelihood tree was constructed using the phylogeny program IQ-TREE2. PCA of the first two principal components was visualized and LD decay was compared between the two groups of rabbits. The genetic structure of the two populations was analyzed with ADMIXTURE [33], with the number of subpopulations (*K* value) ranging from 1 to 5. The *K*-value of 2 had the lowest cross validation error (CV-error). Pi analysis was applied to estimate the degree of variability within each group and the fixation statistic *F*_ST_ was applied to explain population differentiation on the basis of the variance of allele frequencies between the two groups. Both Pi and *F*_ST_ were calculated using a sliding window approach using Vcftools, with a window size of 50 kb and a step size of 10 kb. The candidate signatures of selection that were discovered with the top 5% of Pi and *F*_ST_ were treated as highly divergent windows. Adjacent windows were merged into a single divergent region and annotated.

Functional enrichment analysis was performed by the Database for Annotation, Visualization and Integrated Discovery (DAVID) software to analyze gene ontology (GO) and Kyoto Encyclopedia of Gene and Genome (KEGG) pathways [34]. The P-value for gene set enrichment was corrected using the Benjamini–Hochberg false discovery rate (FDR).

### Estimation of whole-genome SNP-based heritability

We used a three-trait model to analyze wool traits at three time points and a four-trait model for body weight. The three-trait model for the estimation of heritabilities of the studied traits was:

1 $$\mathbf{y}=\left({\mathbf{I}}_{3}\otimes \mathbf{X}\right)\mathbf{b}+\mathbf{u}+\mathbf{e},$$

2 $$\mathrm{where}\,\mathbf{y}=\left[\begin{array}{c}{\mathbf{y}}_{\mathbf{1}}\\ {\mathbf{y}}_{\mathbf{2}}\\ {\mathbf{y}}_{\mathbf{3}}\end{array}\right],\mathbf{u}=\left[\begin{array}{c}{\mathbf{u}}_{\mathbf{1}}\\ {\mathbf{u}}_{\mathbf{2}}\\ {\mathbf{u}}_{\mathbf{3}}\end{array}\right],\mathrm{and }\,\mathbf{e}=\left[\begin{array}{c}{\mathbf{e}}_{\mathbf{1}}\\ {\mathbf{e}}_{\mathbf{2}}\\ {\mathbf{e}}_{\mathbf{3}}\end{array}\right].$$

Here, ${\mathbf{y}}_{i}$ is a vector of phenotypic values for the trait at the $i$th time point; ${\mathbf{b}}_{i}$ is a vector of fixed effects (population mean, sex and rabbit house); ${\mathbf{u}}_{i}$ is a vector of additive polygenic genetic effects; ${\mathbf{e}}_{i}$ is a vector of residual errors; $\mathbf{X}$ is the design matrix for the fixed effects; and $\otimes$ is the Kronecker product. The assumed distributions of the random effects were:

$$\left[\begin{array}{c}{\mathbf{u}}_{\mathbf{1}}\\ {\mathbf{u}}_{\mathbf{2}}\\ {\mathbf{u}}_{\mathbf{3}}\end{array}\right]\sim N({\mathbf{0}}, \sum_{u}\otimes \mathbf{K})$$ and,

3 $$\left[\begin{array}{c}{\mathbf{e}}_{\mathbf{1}}\\ {\mathbf{e}}_{\mathbf{2}}\\ {\mathbf{e}}_{\mathbf{3}}\end{array}\right]\sim N({\mathbf{0}}, \sum_{e}\otimes \mathbf{I}).$$

Here, ${\Sigma }_{u}$ and ${\Sigma }_{e}$ are $3 \times 3$ covariance matrices for the additive polygenic effects and residual errors, respectively, and $\mathbf{K}$ is the genotype-based genomic relationship matrix constructed using the method of VanRaden [35] as:

4 $$\mathbf{K}=\frac{\mathbf{W}\mathbf{W}\mathrm{^{\prime}}}{\sum 2{p}_{j}(1-{p}_{j})},$$

where, $\mathbf{W}$ is the centralized marker genotype matrix with its $ij$th element equal to:

5 $${w}_{ij}={m}_{ij}-2{p}_{j},$$

where ${m}_{ij}$ (2, 1, or 0) is the original genotype of individual $i$ for SNP $j$, and ${p}_{j}$ is the MAF of SNP $j$.The narrow sense heritability for the trait at the $i$th time point was defined as:

6 $${h}_{a,i}^{2}=\frac{{\Sigma }_{u,ii}}{{\Sigma }_{u,ii}+{\Sigma }_{e,ii}},$$

where ${\Sigma }_{u,ii}$ is the additive genetic variance for the $i$th time point, i.e., the $i$th diagonal element of ${\Sigma }_{u}$, and ${\Sigma }_{u,ii}$ is the residual variance for the $i$th time point, i.e., the $i$th diagonal element of ${\Sigma }_{e}$.

The four-trait model was accordingly defined as the three-trait model.

### Multivariate GWAS

To perform multivariate GWAS, the genotype at a single SNP was added as a fixed effect to Eq. (1), resulting in the following model:

7 $$ {\mathbf{y}} = \left( {{\mathbf{I}}_{3}  \otimes {\mathbf{X}}} \right){\mathbf{b}} + \left( {{\mathbf{I}}_{3}  \otimes {\mathbf{w}}} \right){\varvec{\upalpha }} + {\mathbf{u}} + {\mathbf{e}}, $$

with ${\varvec{\upalpha}}={\left[{\alpha }_{1} {\alpha }_{2} {\alpha }_{3}\right]}{\boldsymbol{^{\prime}}}$, where ${\alpha }_{1}$ is the SNP’s allele substitution effect for the trait at the $i$th time point and $\mathbf{w}$ is a vector of SNP genotypes with values of 0, 1 or 2, respectively, for $aa$, $Aa$ and $AA$. The following Wald Chi-square test statistic was computed to test the significance of the SNP effects:

8 $$\left[{\widehat{\alpha }}_{1} {\widehat{\alpha }}_{2} {\widehat{\alpha }}_{3}\right]{\left(\mathrm{var}\left[\begin{array}{c}{\widehat{\alpha }}_{1}\\ {\widehat{\alpha }}_{2}\\ {\widehat{\alpha }}_{3}\end{array}\right]\right)}^{-1}\left[\begin{array}{c}{\widehat{\alpha }}_{1}\\ {\widehat{\alpha }}_{2}\\ {\widehat{\alpha }}_{3}\end{array}\right]\sim {\chi }^{2}\left(3\right).$$

To adjust for multiple testing to control false-positive rates, the threshold for genome-wide significance was 0.05/N, where N is the number of effective SNPs calculated by the PLINK “--indep-pairwise 50 5 0.2” command [36].

### Conditional GWAS

To confirm whether the significant SNPs within clusters of loci are independent or are in high LD, we also performed conditional GWAS with the significant lead SNP (the SNP in the genomic region that has the smallest *P* value) set as a fixed effect in the following model:

9 $$\mathbf{y}=\left({\mathbf{I}}_{3}\otimes \mathbf{X}\right)\mathbf{b}+\left({\mathbf{I}}_{3}\otimes {\mathbf{w}}_{lead}\right){{\varvec{\upalpha}}}_{lead}+\left({\mathbf{I}}_{3}\otimes \mathbf{w}\right){\varvec{\upalpha}}+\mathbf{u}+\mathbf{e},$$

where ${{\varvec{\upalpha}}}_{lead}$ is the effect of the lead SNP and ${\mathbf{w}}_{lead}$ is a vector of SNP genotypes for the lead SNP, ${\varvec{\upalpha}}$ is the test SNP effect (except for the lead SNP) and $\mathbf{w}$ is a vector of the SNP genotypes.

### Estimation of confidence intervals of QTL regions

Confidence intervals of identified QTL regions were estimated by the drop log(*P*) method, following [37], which we expanded to its use for a multivariate linear mixed model. Using the SNP effects estimated with Eq. (5), we first removed the effect of the lead SNP at each QTL from the vector of phenotypes. We then randomly selected 1000 SNPs within the candidate QTL region and assigned the effect of the lead SNP to these selected SNPs, successively. The effects of simulated causal SNPs were added to the above vector of residual phenotypes (removing the lead SNP effect from the original phenotypes), one SNP at a time, to produce 1000 simulated datasets. A local association analysis of the region using Eq. (5) with the simulated phenotype was performed, and the drop in log(*P*) value between the lead SNP for that data set and the simulated causal SNP was recorded. The distribution of these drops in log(*P*) was then estimated across the 1000 simulations, and the 95th percentile was used to determine confidence intervals of the QTL region identified in the original data.

### Estimation of the heritability of QTL

We re-estimated the whole-genome SNP-based heritability adjusted for the lead SNP, ${h}_{a,i}^{{^{\prime}}2}$, by adding the lead SNP to the model, i.e.:

10 $${\mathbf{y}} = \left( {{\mathbf{I}}_{3} \otimes {\mathbf{X}}} \right){\mathbf{b}} + \left( {{\mathbf{I}}_{3} \otimes {\mathbf{w}}_{{lead}} } \right){{\varvec{\upalpha}}}_{lead} + {\mathbf{u}} + {\mathbf{e}},$$

with effects as described previously. The QTL heritability was then estimated as:

11 $${h}_{QTL}^{2}={h}_{a,i}^{2}-{h}_{a,i}^{{{\prime}}2},$$

where ${h}_{a,i}^{2}$ is the whole-genome SNP-based heritability from Eq. (6) without adjustment for the lead SNP effects.

## Results

### LCS imputation pipeline

**Fig. 1:**
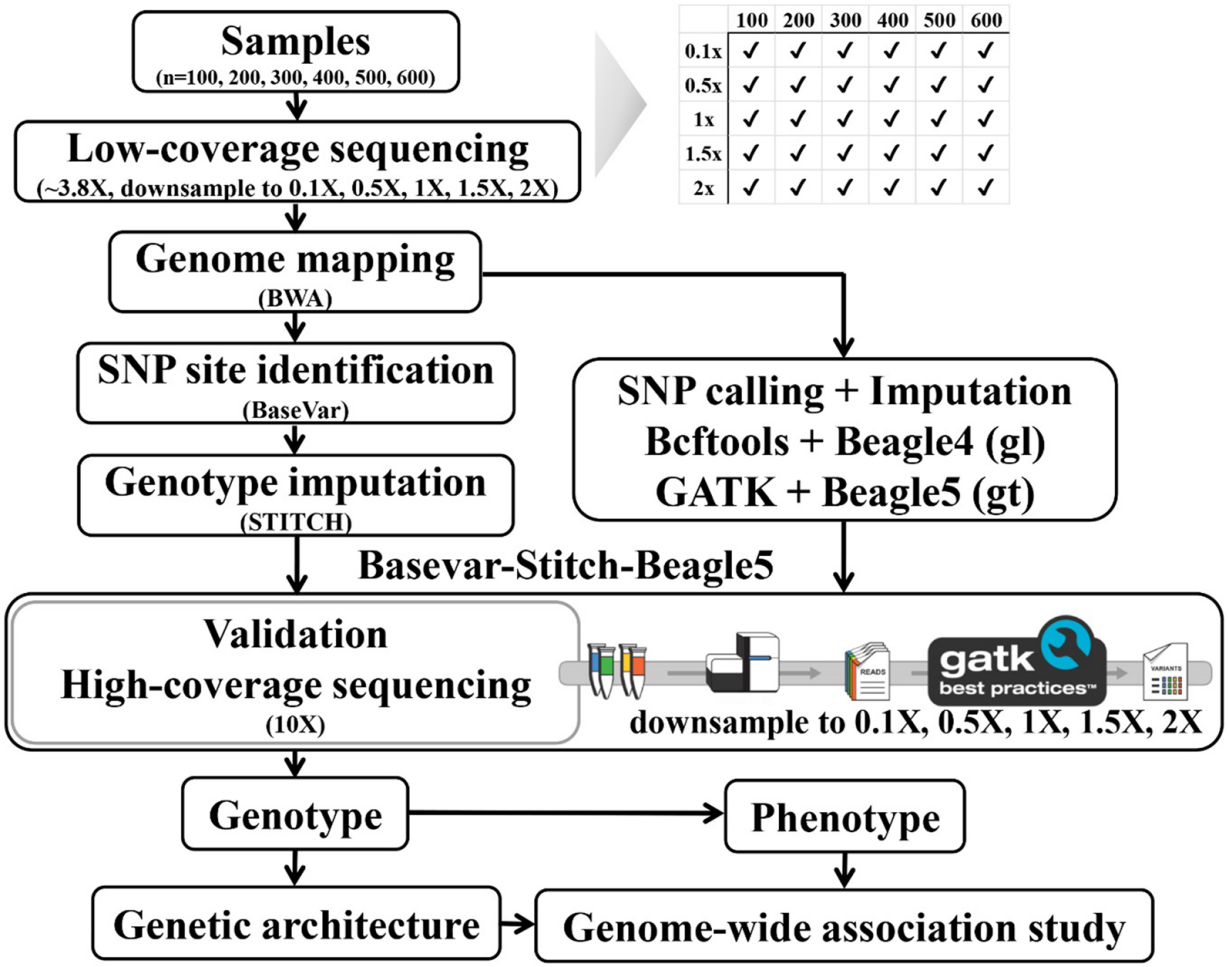
Pipeline for the analysis of low coverage sequence data and genetic architecture

In order to accurately capture variants in the rabbit genome, we compared three genotyping pipelines using LCS data and used the high-depth sequencing data results on chromosome 11 (Chr 11) as the gold standard for accuracy evaluation (Fig. 1). Chromosome 11 was chosen because it has a similar LD extent as the whole genome (see the results on genetic architecture below) and it is a middle-sized chromosome. Variant sites were identified based on all samples using all sequencing data to insure the number of variants. The variant sites discovered by each variant caller (BaseVar, Bcftools and GATK) are shown in a Venn diagram (see Additional file 1: Fig. S1), with BaseVar detecting the largest number of variants. The screening of Chr11 with BaseVar identified 1,737,601 polymorphic sites, which for the most part was also detected by Bcftools and GATK. Bcftools identified 1,710,235 polymorphic sites, of which 1,333,126 overlapped with those identified by BaseVar. GATK identified 1,474,234 polymorphic sites, of which 1,198,453 overlapped with those identified by BaseVar.

**Fig. 2:**
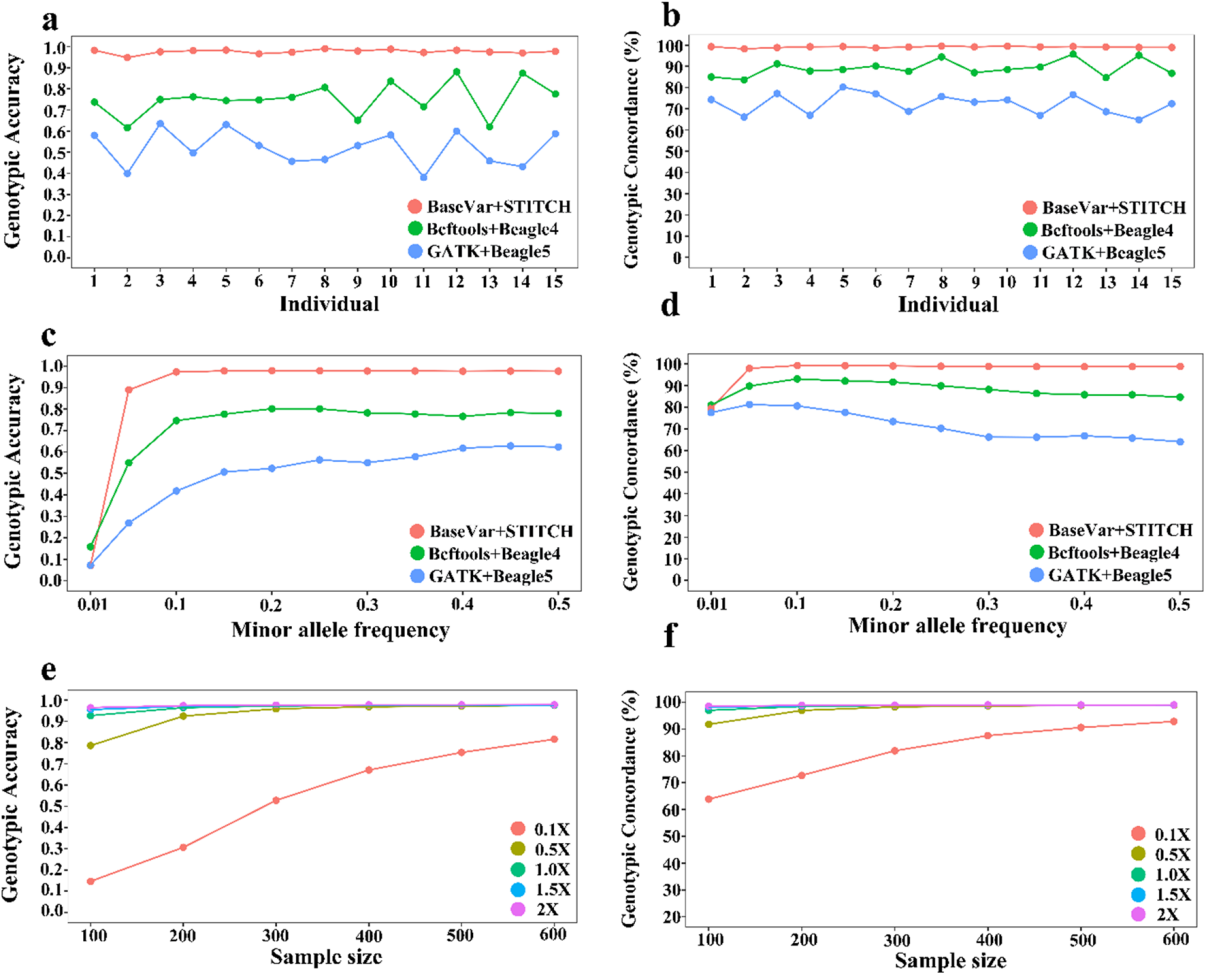
Comparison of the genotype imputation performance between three pipelines (red: BaseVar + STITCH, green: Bcftools + Beagle4 and blue: GATK + Beagle5) and different sample sizes (100, 200, 300, 400, 500 and 600) and sequencing depths (0.1 × , 0.5 × , 1.0 × , 1.5 × , 2.0 ×) based on genotype concordance (on the right) and genotype accuracy (on the left): **a**, **b** for 15 individuals, **c**, **d** for different minor allele frequencies, and **e**, **f** for different sample sizes and sequencing depths

Genotype imputation of the variants was performed for the 600 rabbits with a down-sampled sequencing depth of 2X. Among the reference-panel-free methods, highly accurate genotypes were obtained using the pipeline BaseVar + STITCH, with an average GC of 99.08% and an average GA of 0.98, while for both Bcftools + Beagle4 and GATK + Beagle5, GC did not exceed 95.73%, and GA did not exceed 0.88 (Fig. 2a, b) and (see Additional file 2: Table S1).

We compared the patterns of imputation performance of the three pipelines in relation to MAF. Using the BaseVar + STITCH pipeline, high and stable imputation accuracy (average GC of 98.98% and GC, ranging from 98.82 to 99.31%, and average GA of 0.98 and GA ranging from 0.97 to 0.98) were obtained for common variants with a MAF ranging from 0.05 to 0.5. However, for this MAF range, the Bcftools + Beagle4 pipeline resulted in a poorer imputation accuracy and its accuracy was greatly affected by MAF (GC ranging from 84.66 to 93.10% and GA from 0.75 to 0.80), and using the GATK + Beagle5 pipeline, imputation accuracy was even worse and fluctuated more (GC ranging from 64.02 to 80.60% and GA from 0.42 to 0.63). For SNPs with a MAF lower than 0.05, both GA and GC tended to decrease compared to those with a higher MAF, and were greatly affected by MAF (Fig. 2c, d) and (see Additional file 2: Table S2), which means that the imputation accuracy of rare variants can be highly influenced by MAF. Based on the above results, the best-performing pipeline was BaseVar + STITCH, thus this pipeline was used in the subsequent analyses.

### Effect of sample size and sequencing depth on imputation

As expected, GC and GA generally increased as sample size and sequencing depth increased. In particular, imputation accuracy improved greatly when sample size increased from 100 to 300 and sequence coverage increased from 0.1X to 1.0X. For sequencing depths higher than 1X, sample sizes larger than 300 had little effect on imputation performance, and showed a credible genotyping (Fig. 2e, f) and (see Additional file 2: Table S3).

### Tag SNPs

**Fig. 3:**
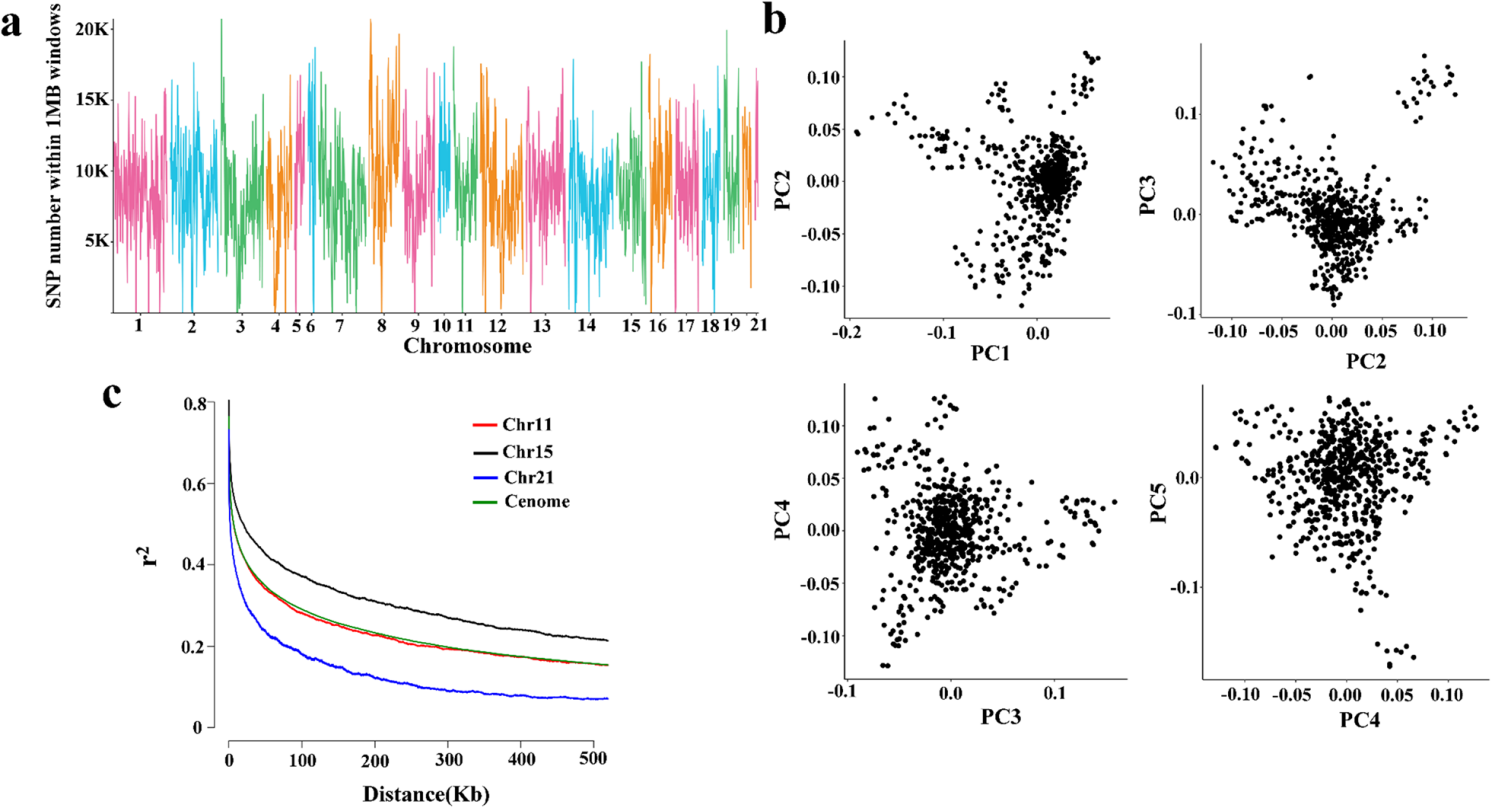
Genetic diversity of the Angora rabbit population. **a** Distribution of SNPs in 1-Mb windows across the genome; **b** Principal component analyses plotting the first to the fifth dimension; and **c** Extent of linkage disequilibrium (LD), values are mean LD r^2^ for all pairs of SNPs binned by distance. The slowest and fastest LD decays were observed for Chr15 and Chr21, respectively. Chr11 showed similar LD extent to that of the whole genome

We retained 18,577,154 high-quality imputed SNPs by two-step imputation using STITCH followed by Beagle and stringent quality control. The SNP density corresponded to 1 SNP per 150 bp in the rabbit genome. The variants were distributed uniformly along the genome (Fig. 3a). The majority of the identified SNPs were located in intergenic (57.78%) and intronic regions (35.50%), with exonic regions containing only 0.52% of the SNPs, which included 72,552 synonymous SNPs and 23,328 nonsynonymous SNPs resulting in a nonsynonymous/synonymous ratio of 0.32 (see Additional file 2: Table S4).

### Genetic architecture

The population structure of the 629 rabbits was assessed by performing PCA. The first five principal components showed no distinct evidence of population structure (Fig. 3b). LD analysis indicated that the average physical distance between adjacent SNP pairs was ~ 6.5 kb (r^2^ = 0.50), Fig. 3c and (see Additional file 2: Table S5). The average pairwise r^2^ decreased to 0.16 and 0.11 for an average distance between SNPs of 500 kb and 1 Mb, respectively. The distribution of r^2^ with respect to physical distance differed by chromosome. The slowest and fastest LD decays were observed for Chr15 and Chr21, respectively. Chr11 showed a level of LD extent that was similar to that of the whole genome. Combining CLR and Pi analyses, we identified 151 potential signatures of selection, which overlapped with 309 candidate genes (see Additional file 1: Fig. S2 and Additional file 2: Table S6). These regions displayed a significant overrepresentation of genes involved in immunity (*P* = 4.10 × 10^–12^) and vitamin B6 metabolism (*P* = 1.30 × 10^–4^) (see Additional file 2: Table S7). The immune system is one of the systems that is strongly targeted by natural selection during evolution because it serves as the backbone of defense against pathogens [38–40]. Vitamin B6 is actively involved as a catalyst in the metabolism of proteins to activate the chemical reactions that initiate the metabolism of the hair proteins, i.e. keratin and melanin, and when these reach a sufficient level in the hair follicles, such that hair growth and renewal are promoted. Clinical studies in humans and mice have shown that vitamin B6 plays a role in improving hair condition and in reducing hair loss [41, 42]. In addition, several genomic regions that are involved in tryptophan, valine, leucine, isoleucine, nicotinate, nicotinamide, tyrosine, and retinol metabolism contained signatures of selection (see Additional file 2: Table S7).

**Fig. 4:**
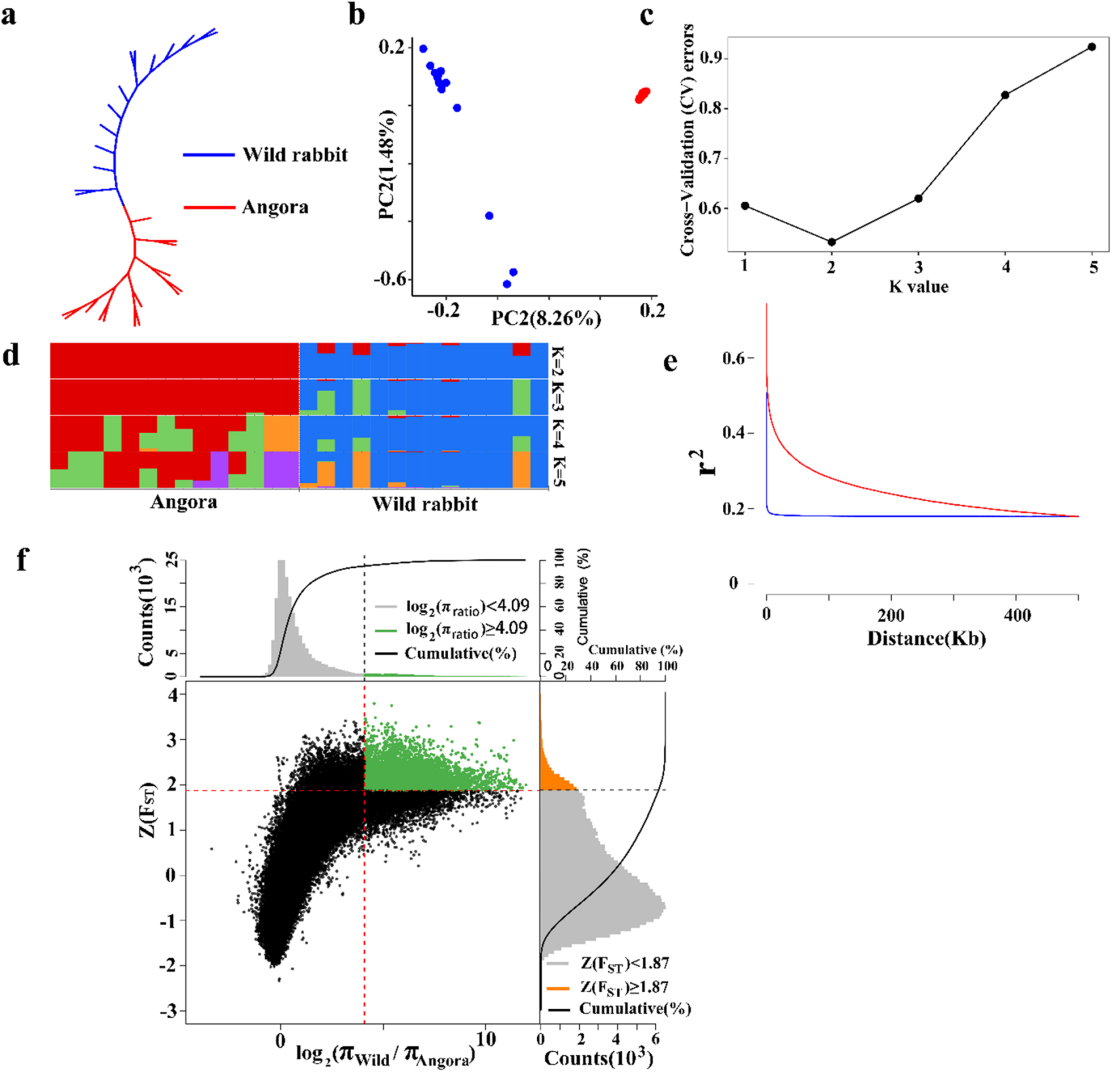
Genetic diversity between the Angora and wild rabbit populations. **a** Maximum-likelihood tree; **b** Principal component analysis showing the genetic differentiation between the two populations; **c** Ancestral population analysis (K = 1 to 5); **d** Admixture plots based on different numbers of assumed ancestors; **e** Extent of LD, values are the mean LD r^2^ for all pairs of SNPs binned by distance. In all the panels, red indicates Angora rabbits and blue wild rabbits. **f** Genomic regions with strong selective sweep signals in Angora and wild rabbits. Distributions of π ratios (wild/Angora) and Z(FST) values were calculated in 50-kb windows with 10-kb steps. Genomic regions under selection during domestication are shown as green points located to the top-right regions that correspond to the 5% right tails of empirical log2 (πwild/πAngora) ratio distribution and the top 5% empirical Z(FST) distribution. The vertical and horizontal gray lines represent the top 5% value of log2 (πwild/πAngora) (4.09) and Z(FST) (1.87), respectively

For genetic diversity between the Angora and wild rabbit populations, a maximum-likelihood tree showed that the genotypes could be classified into the two obvious divergent groups (Fig. 4a). The PCA showed diversity among the rabbit genotypes, with the first two principal components explaining 8.26% and 1.48% of the genetic variance, respectively (Fig. 4b). All individuals from the Angora rabbit population were grouped together and showed a consistent genetic relationship that coincided with that of rabbits artificially inseminated with mixed semen, while individuals of the wild rabbit population were relatively dispersed, probably because they originated from different geographical regions. In addition, assessment of the population structure using *K* values ranging from 1 to 5 showed that the most significant change of likelihood of the population number occurred when *K* increased from 1 to 2 (Fig. 4c) and thus, the most likely value of *K* was 2. Compared to *K* = 3, 4, or 5, at *K* = 2, the two populations clearly separated from each other, which indicates that their genetic backgrounds are significantly different. Such partitioning of these populations was consistent with the ancestral population analysis using *K* values (Fig. 4d) and was also in accordance with the maximum-likelihood tree (Fig. 4a). LD was calculated to provide information on population history. LD between markers decreased as the physical distance between markers increased, but the degree of LD attenuation with distance differed greatly between the two populations. The wild population exhibited an extremely rapid LD decay, indicating the high diversity of the wild ancestors. In contrast, the Angora population showed a slow decay of LD, and the average r^2^ was higher than 0.2 for markers separated by 350 kb, which indicates high inbreeding that could be due to intense artificial selection (Fig. 4e) and (see Additional file 2: Table S8).

Using the top 5% of *F*_ST_ values and *θ*_*π*_ ratios (cutoffs: *F*_ST_ > 1.87 and log_2_ (*θ*_*π*_ ratio (*θ*_*π* wild_/*θ*_*π* Angora_) ≥ 4.09)), we identified 464 candidate domestication regions that overlapped with 775 genes under selection in the domestic rabbit (Fig. 4f) and (see Additional file 2: Tables S9, S10). Gene set enrichment analysis highlighted genes that were mainly involved in the nervous system, probably reflecting the effect of domestication on tameness and aggression [43]. In addition, 155 regions were identified in both the within-breed and the across-breed signatures of selection, which overlapped with 354 genes (see Additional file 2: Tables S11, S12). Gene set enrichment analysis of these genes highlighted genes that were mainly involved in metabolism, immunity, and the nervous system.

### Genome-wide association analyses

**Fig. 5:**
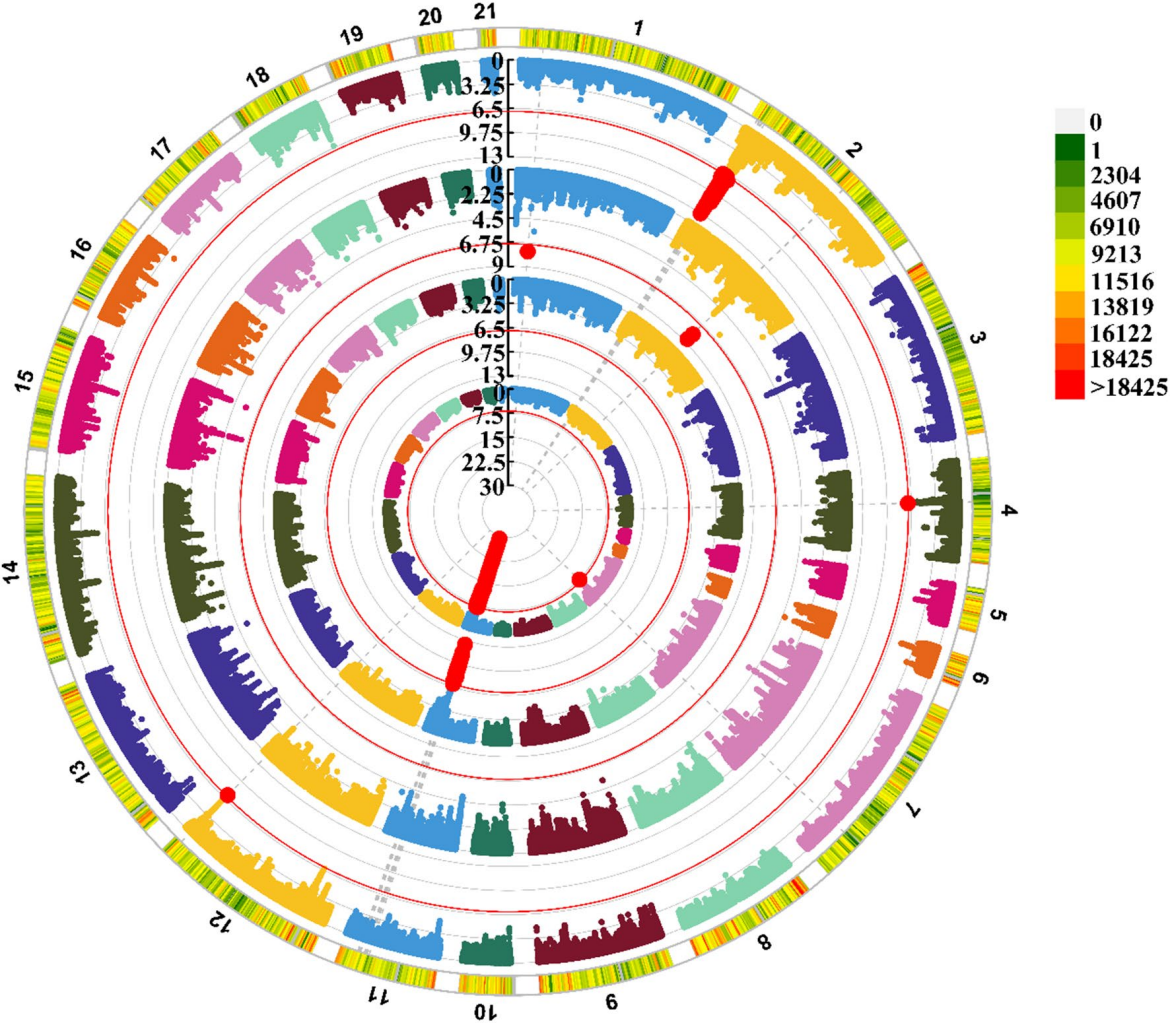
Circle Manhattan plot showing the associations between SNPs and wool traits: diameter of fine wool (DFW), coefficient of variation of diameter of fine wool (CVDFW), length of fine wool (LFW) and body weight (BW), respectively (from inside to outside the circle), in the Angora rabbit population. The threshold lines indicate the genome-wide significance level (−log_10_(0.05/391,976)) after Bonferroni correction

Phenotypes of six traits (LFW, DFW, CVDFW, LCW, RCW, and BW) and genotypes on up to 18,577,154 autosomal SNPs after imputation were available for the 629 rabbits. For association testing, we used a multivariate linear mixed model, as implemented in our software GMAT (https://github.com/chaoning/GMAT). After LD-based pruning with PLINK, 391,976 independent SNPs were in approximate linkage equilibrium with each other and, thus, the genome-wide significance level was 1.28 × 10^–9^ after Bonferroni correction. Significant SNPs that were found to be associated with DFW, CVDFW, LFW, and BW are in Tables S13–S16 (see Additional file 2: Tables S13–S16). A circle Manhattan plot of the significant associations between SNPs and four traits (DFW, CVDFW, LFW and BW) is in Fig. 5. The quantile–quantile plots for each trait are in Figs. S3 and S4 (see Additional file 1 Figs. S3, S4). In summary, six (five non-overlapping) QTL were identified and six independent top significant SNPs were located for the CVDFW, DFW, and BW traits (Table 1). No QTL were identified for LFW, LCW, or RCW.

**Table 1:** QTL mapping and the 95% confidence interval (95%CI) of each QTL

Trait	QTL	Chr	95% CI		95% CI width	Top SNP	Allele 0	Allele 1	MAF	*P*	Number of annotated genes^a^
DFW	QTL1	7	138,910,411	139,598,335	687,925	138,975,863	T	C	0.053963	1.36 × 10^–7^	2
	QTL2	11	64,944,841	64,952,273	7433	64,951,174	G	C	0.071669	7.12 × 10^–23^	2
CVDFW	QTL1	11	64,270,630	65,432,659	1,162,030	65,412,003	A	T	0.079723	1.42 × 10^–11^	6
BW	QTL1	2	8,257,045	8,923,024	665,980	8,877,739	G	A	0.174952	7.33 × 10^–12^	4
	QTL2	4	36,704,911	38,161,753	1,456,843	38,127,528	A	G	0.083174	5.93 × 10^–7^	61
	QTL3	12	148,360,582	148,642,693	282,112	148,369,145	G	A	0.083174	4.61 × 10^–7^	3

^a^QTL for DFW contained no genes, and the nearest genes in the flanking regions were annotated

Conditional GWAS was applied with significant lead SNPs fitted as fixed covariates, but no additional significant SNPs were detected, which indicates that all the significant SNPs in each QTL were in LD with the most significant SNP and the causal SNP might be at or nearby the latter. To assist gene identification, the 95% confidence interval (95% CI) of each QTL was estimated, which showed an average width of 0.71 Mb (7.43 kb to 1.46 Mb), with 67% of the QTL having an average CI width less than 1 Mb (Table 1).

### Heritability estimates based on genome-wide SNPs and by QTL

SNP-based heritability was estimated for the six traits and ranged from 7.5 to 39.1%, which indicates a low to medium heritability, with a mean value of 19.0% (see Additional file 2: Table S17). Among these six traits, the heritability estimate was highest for BW and lowest for CVDFW. The heritability explained by the detected QTL was analyzed by fitting the most significant SNPs located in these QTL as fixed effects. The QTL associated with the three traits explained 2.28 to 8.52% of the phenotypic variation (see Additional file 2: Table S18). Among these, DFW exhibited the strongest QTL effect, with 7.50% of the phenotypic variation explained by QTL2. CVDFW showed a low QTL effect, similar to its SNP-based heritability.

### Identification of candidate genes within the identified QTL

The number of annotated genes covered by the QTL regions (based on 95% CI) ranged from 0 to 61, with a mean of 12 (Table 1) and (see Additional file 2: Table S19). Among these, three QTL regions contained less than 10 genes. Two QTL for DFW did not overlap with any gene because of their small 95% CI, thus we searched for the upstream and downstream genes that were nearest to the 95% CI, which were 243,754 and 279,302 bp away from the QTL1 interval and 47 4,777 and 37,659 bp away from the QTL2 interval. We focused on QTL that contained a small number of genes because they provided a starting point for functional investigations. For rabbit wool traits, the QTL for CVDFW on Chr11 contained six genes, among which the *fibroblast growth factor 10* (*FGF10*) gene was most relevant. The most significant locus (Chr11: 65,412,003 bp, *P* = 1.42E×10^−11^) associated with fine fiber was detected closest to the *FGF10* gene (Chr11: 64,989,932–65,080,400), which was also the nearest gene to the QTL for DFW (Chr11: 64,944,841–64,952,273). The *FGF10* gene is a member of the fibroblast growth factor (FGF) gene family that possesses broad mitogenic and cell survival activities and is well-known for its role in the regulation of hair morphogenesis and hair growth cycle in humans and mice [44, 45].

The QTL for body weight located on Chr2 and Chr12 contained four and three genes, respectively. For several of these genes, associations with traits have been previously reported: *FAM184B* with chicken body weight [46, 47]; the region containing the *DCAF16* and *NCAPG* genes with average daily gain in cattle based on a multi-strategy GWAS [48]; the region containing the *NCAPG* and *LCORL* genes is known for its association with human height and with body weight/height in horses and cattle and has also been shown to harbor a signature of selection in pigs and dogs [49].

## Discussion

Wool traits are important in rabbits, because their fur is one of the most preferred natural fibers in the textile industries. A well-known breed for fiber production is the Angora rabbit and the fibers obtained from its wool are usually chosen for the production of luxury textile materials. In addition, the rabbit was the first and has since long been used as a model to dissect the genetic architecture of human diseases [50], and also as a model to study hair growth, although most studies on hair growth have been performed on humans, sheep, and mice [51–54]. In this study, we focused on several wool characteristics, such as fiber diameter and length, that are essential in wool rabbit breeding, as well as important indicators of the spinning efficiency of the wool. To dissect the genetic architecture of complex wool traits in Angora rabbits, we first investigated high-density SNPs that were derived in a cost-effective manner using ultra-low coverage whole-genome sequencing, combined with three genotype imputation strategies, using different levels of sequencing coverage and sample sizes to insure imputation performance. Then, we performed GWAS and conditional GWAS with a multivariate linear mixed model and mapped QTL to 95% confidence intervals to identify candidate genes at a high resolution.

Performing genotype imputation across the whole genome boosts the number of detected SNPs and has been used widely in GWAS to provide a high-resolution view of associated regions, and to increase the chance of directly identifying causal SNPs for QTL [55]. Obtaining genotyping information by combining LCS with genotype imputation has several advantages due to the decreasing costs of both DNA library construction and sequencing [17, 37], especially when dense microarrays are lacking [11, 56]. This approach has been shown to result in increased performance in a number of scenarios with different study designs. We compared several variant imputation pipelines. Beagle was tested to determine whether it was suitable for LCS data resulting from the conventional SNP calling software Bcftools/GATK. STITCH and its ally BaseVar were applied in this study greatly outperformed the other pipelines, as expected. As a proof of principle, we imputed genotypes for five levels of LCS coverages in overlapping samples of six sizes to assess how sequencing depth and sample size influence imputation power. We found that sample size greatly affected imputation accuracy at an ultra-low sequencing coverage (< 1.0X). At a sequencing depth of 1.0X, BaseVar + STITCH reached a high imputation accuracy with a GC > 98.84% and GA > 0.97 when sample size was larger than 300. The patterns of imputation accuracy obtained in this study with the different pipelines and with different levels of sequencing depth and sample sizes are in line with results from previous studies [11, 26, 57, 58].

Compared to the use of SNP chips, LCS avoids the problem caused by the ascertainment bias of common arrays, captures genetic variation in an unbiased manner, identifies novel variants, and enhances variant discovery, particularly in underrepresented populations [8]. The cost of LCS at a sequencing depth of 1X is comparable to and can even be lower than that of SNP arrays. In addition, in spite of the decrease in unit sequencing cost, the cost of high-depth sequencing of a large population remains high. Compared to high-coverage sequencing, LCS can save genotyping costs per sample by a factor of more than 10, while providing enough information, which makes it applicable for sequencing larger samples. Therefore, we strongly recommend low coverage sequencing combined with genotype imputation as a cost-effective and powerful alternative to SNP arrays and high-depth sequencing for more powerful genetic analyses.

Using a large sample size and a high-resolution genome screen, recombination events can be detected to accurately identify causal variants that underlie a quantitative trait [6, 7]. In our study, by using whole-genome SNPs that were identified by LCS combined with genotype imputation in a population of 629 rabbits, we discovered six QTL associated with growth and wool traits that explained from 0.42 to 7.50% of the phenotypic variation. After fine mapping, we focused on the *FGF10* gene for its association with fiber growth and diameter. Several members of the FGF gene family, including *FGF10*, are known to be involved in the regulation of the hair growth cycle, mainly by promoting hair follicle (HF) telogen anagen transition by providing the stimulatory signals to the HF stem cells and/or their progenies that reside in the HF bulge and secondary hair germ [44, 59, 60]. In addition, FGF genes may play an important role in hair morphogenesis. For example, *FGF2* [61], *FGF7* [62], *FGF9* [45], and *FGF10* [63] have been shown to contribute to differences in fiber diameter in human and mice. In addition, the FGF7 and FGF10 proteins efficiently and specifically bind to FGFR2-IIIb, which is one of several diverse protein variants with distinct binding characteristics that are encoded by the *FGFR2* gene. Transgenic mice that are deficient for *FGFR2-IIIb* suffer from abnormally thin hairs that are characterized by single columns of medulla cells [63].

Response to strong artificial selection results in standing genetic variation and even in completely fixed mutations across many genomic regions, which reflects the long-term directional selection history for wool traits of the Angora rabbit population and probably explains that few QTL are detected for fiber traits. The LD decay and quantile–quantile plots reflect the strong artificial selection in the Angora rabbit population. LD extends over a long distance in the Angora rabbit genome, in which markers separated by 300 kb have average r^2^ higher than 0.2. The quantile–quantile plots showed the deviation of the observed *P* values from the expected values. In addition, the fact that few QTL were detected for the other fiber traits might be due to their polygenic genetic architecture, i.e., many individual mutations each with a small effect contributing to the total genetic variation and not large enough to reach the significance level when testing for a typical complex trait.

## Conclusions

Low coverage sequencing combined with genotype imputation allows accurate high-density genotypes, even without a good reference panel. GWAS based on LCS data enables QTL detection and fine-mapping of genes associated with quantitative traits. This study provides a cost-effective analysis pipeline that can contribute to unravel the genetic architecture of complex traits and to increase genetic progress in livestock.

## Supplementary Information

**Additional file 1:** **Figure S1.** Venn diagram illustrating the number of the variants called by BaseVar, GATK and Bcftools**. Figure S2.** CLR and Pi analyses in the Angora rabbit population. **Figure S3.** Q-Q plots for the Angora rabbits. **Figure S4.** Manhattan plots for the Angora rabbits.

**Additional file 2: Table S1.** Genotype accuracy and concordance of imputation obtained with the three pipelines on 15 individuals**. Table S2.** Genotype accuracy and concordance of imputation by the three pipelines with the MAF ranges**. Table S3.** Genotype accuracy and concordance of imputation with different sample size and depth by BaseVar + STITCH**. Table S4.** Variant analysis by regions and functions**. Table S5.** LD decay in the Angora rabbit population**. Table S6.** Genes located in the selected regions in the Angora rabbit population**. Table S7.** Enrichment of genes located in the selected regions in the Angora rabbit population**. Table S8.** LD decay in the Angora and wild rabbit populations. **Table S9.** Genes located in the selected regions between Angora and wild rabbits. **Table S10.** Enrichment of genes located in the selected regions between Angora and wild rabbits. **Table S11.** Genes located in the common regions between within-breed and cross-breed signatures of selection. **Table S12.** Enrichment of genes located in the common regions between within-breed and cross-breed signatures of selection. **Table S13.** Significant SNPs for DFW. **Table S14.** Significant SNPs for CVDFW. **Table S15.** Significant SNPs for LFW. **Table S16.** Significant SNPs for BW. **Table S17.** SNP-based heritability. **Table S18.** QTL contribution to phenotypic variance. **Table S19.** Genes overlapping with by QTL.

## Data Availability

The sequencing data used for analysis is available at NCBI (PRJNA810279). SNP-based heritability, GWAS, conditional GWAS, confidence interval and QTL heritability estimation were analyzed with our self-written software available at https://github.com/chaoning/GMAT.

## References

[1] Huang H, Fang M, Jostins L, Umicevic Mirkov M, Boucher G, Anderson CA (2017). Fine-mapping inflammatory bowel disease loci to single-variant resolution. Nature.

[2] da Silva XG, Bellomo EA, McGinty JA, French PM, Rutter GA (2013). Animal models of GWAS-identified type 2 diabetes genes. J Diabetes Res.

[3] Freebern E, Santos DJA, Fang L, Jiang J, Parker Gaddis KL, Liu GE (2020). GWAS and fine-mapping of livability and six disease traits in Holstein cattle. BMC Genomics.

[4] Qin P, Lu H, Du H, Wang H, Chen W, Chen Z (2021). Pan-genome analysis of 33 genetically diverse rice accessions reveals hidden genomic variations. Cell.

[5] Kainer D, Padovan A, Degenhardt J, Krause S, Mondal P, Foley WJ (2019). High marker density GWAS provides novel insights into the genomic architecture of terpene oil yield in Eucalyptus. New Phytol.

[6] Loos RJF (2020). 15 years of genome-wide association studies and no signs of slowing down. Nat Commun.

[7] Ros-Freixedes R, Gonen S, Gorjanc G, Hickey JM (2017). A method for allocating low-coverage sequencing resources by targeting haplotypes rather than individuals. Genet Sel Evol.

[8] Martin AR, Atkinson EG, Chapman SB, Stevenson A, Stroud RE, Abebe T (2021). Low-coverage sequencing cost-effectively detects known and novel variation in underrepresented populations. Am J Hum Genet.

[9] Gilly A, Ritchie GR, Southam L, Farmaki AE, Tsafantakis E, Dedoussis G (2016). Very low-depth sequencing in a founder population identifies a cardioprotective APOC3 signal missed by genome-wide imputation. Hum Mol Genet.

[10] Gilly SA, Southam L, Suveges D, Kuchenbaecker K, Moore R, Melloni GEM (2019). Very low-depth whole-genome sequencing in complex trait association studies. Bioinformatics.

[11] Davies RW, Flint J, Myers S, Mott R (2016). Rapid genotype imputation from sequence without reference panels. Nat Genet.

[12] Browning SR, Browning BL (2007). Rapid and accurate haplotype phasing and missing-data inference for whole-genome association studies by use of localized haplotype clustering. Am J Hum Genet.

[13] Spiliopoulou A, Colombo M, Orchard P, Agakov F, McKeigue P (2017). GeneImp: fast imputation to large reference panels using genotype likelihoods from ultralow coverage sequencing. Genetics.

[14] Rubinacci S, Ribeiro DM, Hofmeister RJ, Delaneau O (2021). Efficient phasing and imputation of low-coverage sequencing data using large reference panels. Nat Genet.

[15] Wasik K, Berisa T, Pickrell JK, Li JH, Fraser DJ, King K (2021). Comparing low-pass sequencing and genotyping for trait mapping in pharmacogenetics. BMC Genomics.

[16] Liu S, Huang S, Chen F, Zhao L, Yuan Y, Francis SS (2018). Genomic analyses from non-invasive prenatal testing reveal genetic associations, patterns of viral infections, and Chinese population history. Cell.

[17] Meier JI, Salazar PA, Kucka M, Davies RW, Dreau A, Aldas I (2021). Haplotype tagging reveals parallel formation of hybrid races in two butterfly species. Proc Natl Acad Sci USA.

[18] Bolger AM, Lohse M, Usadel B (2014). Trimmomatic: a flexible trimmer for Illumina sequence data. Bioinformatics.

[19] Li H, Durbin R (2009). Fast and accurate short read alignment with Burrows-Wheeler transform. Bioinformatics.

[20] McKenna A, Hanna M, Banks E, Sivachenko A, Cibulskis K, Kernytsky A (2010). The genome analysis toolkit: a MapReduce framework for analyzing next-generation DNA sequencing data. Genome Res.

[21] Ros-Freixedes R, Battagin M, Johnsson M, Gorjanc G, Mileham AJ, Rounsley SD (2018). Impact of index hopping and bias towards the reference allele on accuracy of genotype calls from low-coverage sequencing. Genet Sel Evol.

[22] Li H (2011). A statistical framework for SNP calling, mutation discovery, association mapping and population genetical parameter estimation from sequencing data. Bioinformatics.

[23] Browning BL, Browning SR (2016). Genotype imputation with millions of reference samples. Am J Hum Genet.

[24] Browning BL, Zhou Y, Browning SR (2018). A one-penny imputed genome from next generation reference panels. Am J Hum Genet.

[25] Ramnarine S, Zhang J, Chen LS, Culverhouse R, Duan WM, Hancock DB (2015). When does choice of accuracy measure alter imputation accuracy assessments. PLoS ONE.

[26] Teng J, Zhao C, Wang D, Chen Z, Tang H, Li J (2022). Assessment of the performance of different imputation methods for low-coverage sequencing in Holstein cattle. J Dairy Sci.

[27] Chang CC, Chow CC, Tellier LC, Vattikuti S, Purcell SM, Lee JJ (2015). Second-generation PLINK: rising to the challenge of larger and richer datasets. Gigascience.

[28] Wang K, Li M, Hakonarson H (2010). ANNOVAR: functional annotation of genetic variants from high-throughput sequencing data. Nucleic Acids Res.

[29] Yang J, Lee SH, Goddard ME, Visscher PM (2011). GCTA: a tool for genome-wide complex trait analysis. Am J Hum Genet.

[30] Zhang C, Dong SS, Xu JY, He WM, Yang TL (2019). PopLDdecay: a fast and effective tool for linkage disequilibrium decay analysis based on variant call format files. Bioinformatics.

[31] Pavlidis P, Zivkovic D, Stamatakis A, Alachiotis N (2013). SweeD: likelihood-based detection of selective sweeps in thousands of genomes. Mol Biol Evol.

[32] Danecek P, Auton A, Abecasis G, Albers CA, Banks E, DePristo MA (2011). The variant call format and VCFtools. Bioinformatics.

[33] Alexander DH, Novembre J, Lange K (2009). Fast model-based estimation of ancestry in unrelated individuals. Genome Res.

[34] da Huang W, Sherman BT, Lempicki RA (2009). Systematic and integrative analysis of large gene lists using DAVID bioinformatics resources. Nat Protoc.

[35] VanRaden PM (2008). Efficient methods to compute genomic pPredictions. J Dairy Sci.

[36] Purcell S, Neale B, Todd-Brown K, Thomas L, Ferreira MA, Bender D (2007). PLINK: a tool set for whole-genome association and population-based linkage analyses. Am J Hum Genet.

[37] Nicod J, Davies RW, Cai N, Hassett C, Goodstadt L, Cosgrove C (2016). Genome-wide association of multiple complex traits in outbred mice by ultra-low-coverage sequencing. Nat Genet.

[38] Quintana-Murci L (2019). Human immunology through the lens of evolutionary genetics. Cell.

[39] Barreiro LB, Quintana-Murci L (2020). Evolutionary and population (epi)genetics of immunity to infection. Hum Genet.

[40] Gerardo NM, Hoang KL, Stoy KS (2020). Evolution of animal immunity in the light of beneficial symbioses. Philos Trans R Soc Lond B Biol Sci.

[41] Brzezinska-Wcislo L (2001). Evaluation of vitamin B6 and calcium pantothenate effectiveness on hair growth from clinical and trichographic aspects for treatment of diffuse alopecia in women. Wiad Lek.

[42] D'Agostini F, Fiallo P, Pennisi TM, De Flora S (2007). Chemoprevention of smoke-induced alopecia in mice by oral administration of l-cystine and vitamin B6. J Dermatol Sci.

[43] Carneiro M, Rubin CJ, Di Palma F, Albert FW, Alfoldi J, Martinez Barrio A (2014). Rabbit genome analysis reveals a polygenic basis for phenotypic change during domestication. Science.

[44] Greco V, Chen T, Rendl M, Schober M, Pasolli HA, Stokes N (2009). A two-step mechanism for stem cell activation during hair regeneration. Cell Stem Cell.

[45] Kinoshita-Ise M, Tsukashima A, Kinoshita T, Yamazaki Y, Ohyama M (2020). Altered FGF expression profile in human scalp-derived fibroblasts upon WNT activation: implication of their role to provide folliculogenetic microenvironment. Inflamm Regen.

[46] Fan QC, Wu PF, Dai GJ, Zhang GX, Zhang T, Xue Q (2017). Identification of 19 loci for reproductive traits in a local Chinese chicken by genome-wide study. Genet Mol Res.

[47] Zhang GX, Fan QC, Wang JY, Zhang T, Xue Q, Shi HQ (2015). Genome-wide association study on reproductive traits in Jinghai Yellow Chicken. Anim Reprod Sci.

[48] Zhang WG, Li JY, Guo Y, Zhang LP, Xu LY, Gao X (2016). Multi-strategy genome-wide association studies identify the DCAF16-NCAPG region as a susceptibility locus for average daily gain in cattle. Sci Rep.

[49] Takasuga A (2016). *PLAG1* and *NCAPG-LCORL* in livestock. Anim Sci J.

[50] Esteves PJ, Abrantes J, Baldauf H, BenMohamed L, Chen Y, Christensen N (2018). The wide utility of rabbits as models of human diseases. Exp Mol Medi.

[51] Zhao B, Luo H, He J, Huang X, Chen S, Fu X (2021). Comprehensive transcriptome and methylome analysis delineates the biological basis of hair follicle development and wool-related traits in Merino sheep. BMC Biol.

[52] Plowman JE, Harland DP, Campos AMO, Rocha ESS, Thomas A, Vernon JA (2020). The wool proteome and fibre characteristics of three distinct genetic ovine breeds from Portugal. J Proteomics.

[53] Chai M, Jiang MS, Vergnes L, Fu XD, de Barros SC, Doan NB (2019). Stimulation of hair growth by small molecules that activate autophagy. Cell Rep.

[54] Gur-Cohen S, Yang H, Baksh SC, Miao Y, Levorse J, Kataru RP (2019). Stem cell-driven lymphatic remodeling coordinates tissue regeneration. Science.

[55] Marchini J, Howie B (2010). Genotype imputation for genome-wide association studies. Nat Rev Genet.

[56] Davies RW, Kucka M, Su DW, Shi SN, Flanagan M, Cunniff CM (2021). Rapid genotype imputation from sequence with reference panels. Nat Genet.

[57] Yang R, Guo X, Zhu D, Tan C, Bian C, Ren J (2021). Accelerated deciphering of the genetic architecture of agricultural economic traits in pigs using a low-coverage whole-genome sequencing strategy. GigaScience..

[58] Zhao C, Teng J, Zhang X, Wang D, Zhang X, Li S (2021). Towards a cost-effective implementation of genomic prediction based on low cverage whole genome sequencing in Dezhou donkey. Front Genet.

[59] Lin WH, Xiang LJ, Shi HX, Zhang J, Jiang LP, Cai PT (2015). Fibroblast growth factors stimulate hair growth through β-catenin and Shh expression in C57BL/6 mice. BioMed Res Int.

[60] Mardaryev AN, Ahmed MI, Vlahov NV, Fessing MY, Gill JH, Sharov AA (2010). Micro-RNA-31 controls hair cycle-associated changes in gene expression programs of the skin and hair follicle. FASEB J.

[61] Takabayashi Y, Nambu M, Ishihara M, Kuwabara M, Fukuda K, Nakamura S (2016). Enhanced effect of fibroblast growth factor-2-containing dalteparin/protamine nanoparticles on hair growth. Clin Cosmet Investig Dermatol.

[62] Seo HS, Lee DJ, Chung JH, Lee CH, Kim HR, Kim JE (2016). Hominis Placenta facilitates hair re-growth by upregulating cellular proliferation and expression of fibroblast growth factor-7. BMC Complement Altern Med.

[63] Schlake T (2005). FGF signals specifically regulate the structure of hair shaft medulla via IGF-binding protein 5. Development.

