# Supplementary material for: Cost-effectively dissecting the genetic architecture of complex wool traits in rabbits by low-coverage sequencing
Source: Genet Sel Evol. 2022 Nov 18;54:75. doi: 10.1186/s12711-022-00766-y (PMC9673297; doi:10.1186/s12711-022-00766-y)

**Fig. S1.** Venn diagram illustrating the number of the variants called by BaseVar, GATK and Bcftools


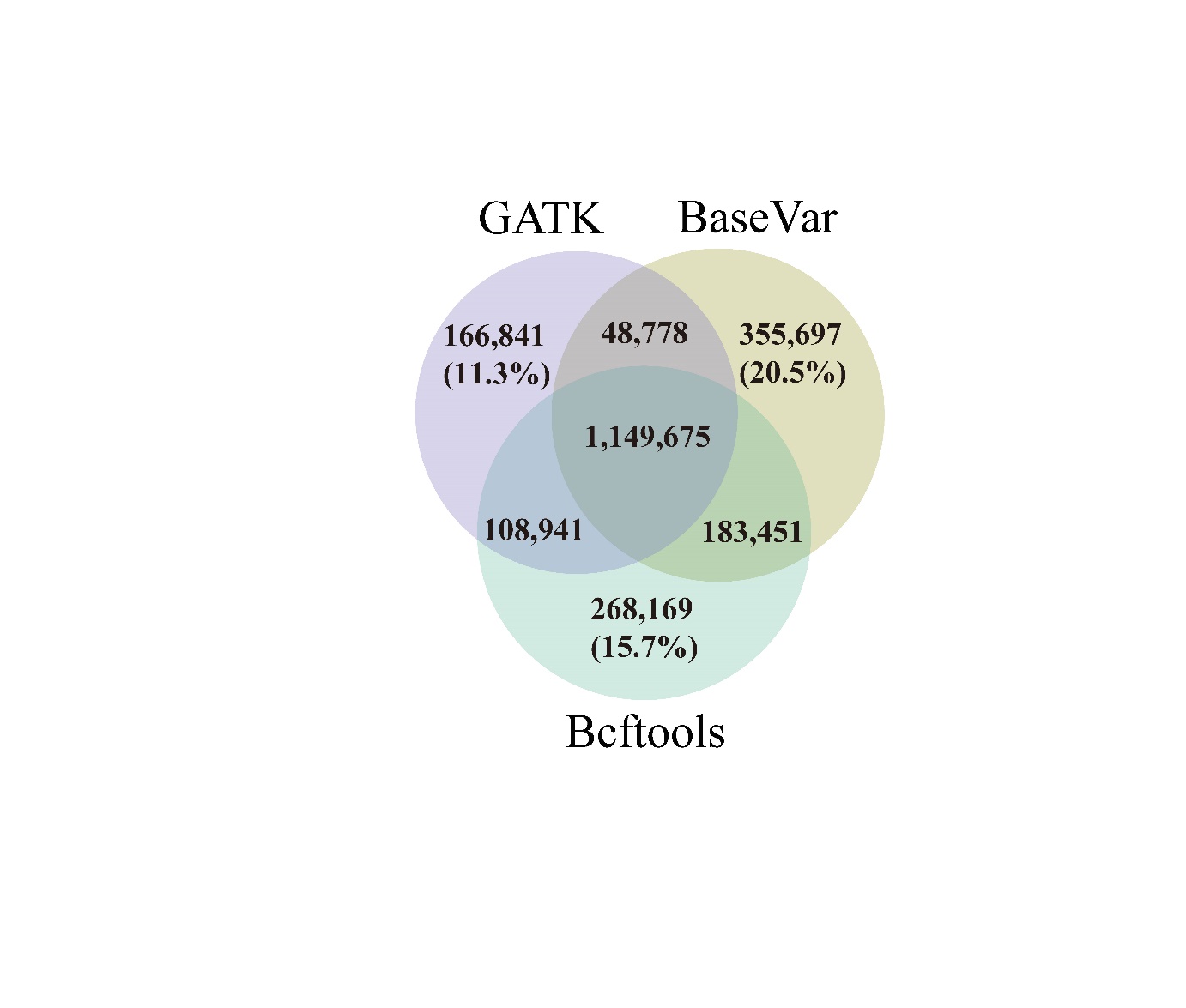


**Fig. S2.** CLR and Pi analyses in the Angora rabbit population


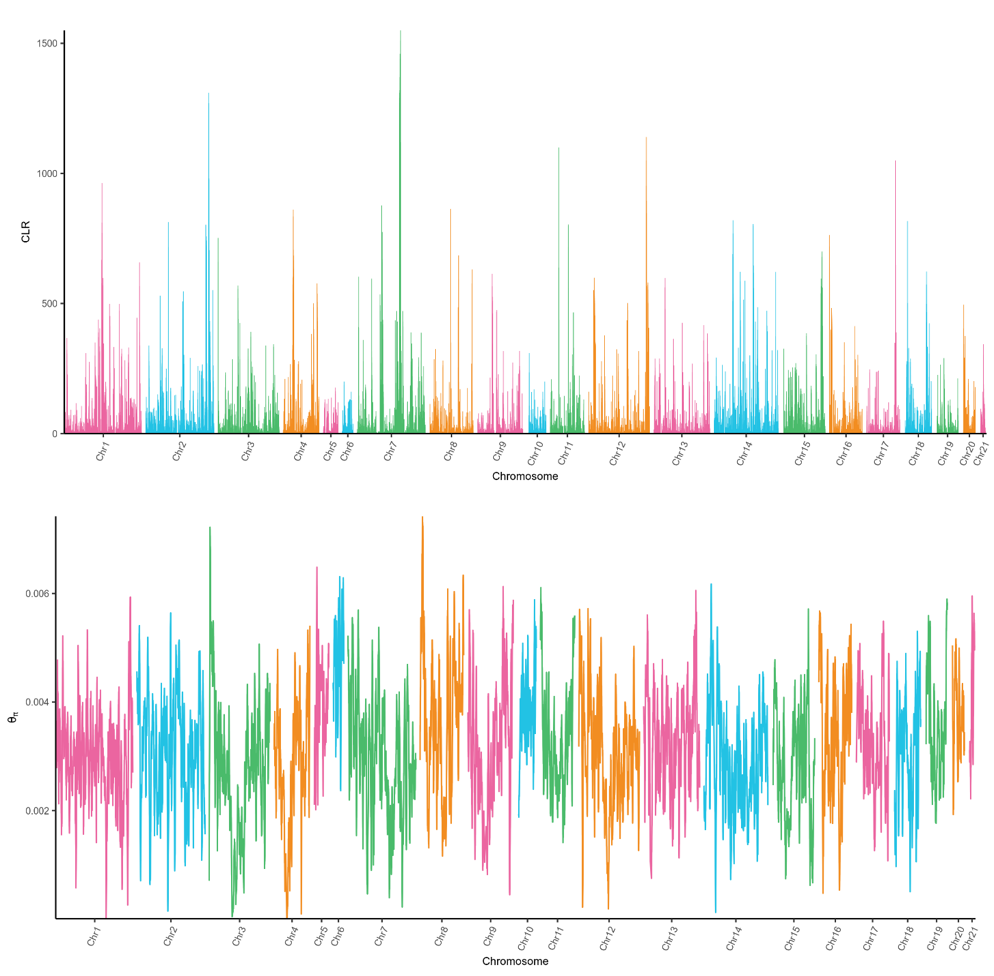


**Fig. S3.** Q-Q plots for the Angora rabbits (A: Body weight, B: DFW, C: CVDFW, D: LCW, E: LFW, F: RCW)


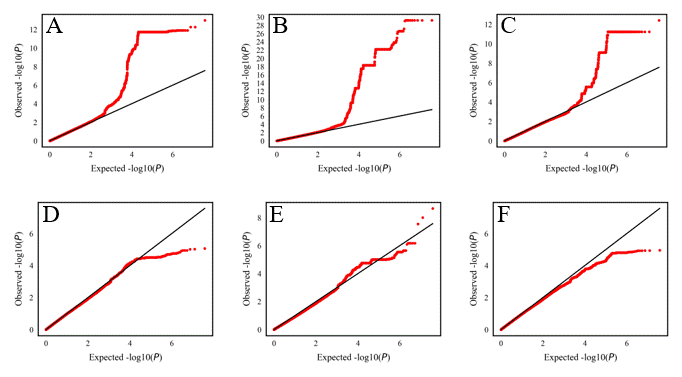


**Fig. S4.** Manhattan plots for the Angora rabbits (A: Body weight, B: DFW, C: CVDFW, D: LCW, E: LFW, F: RCW)


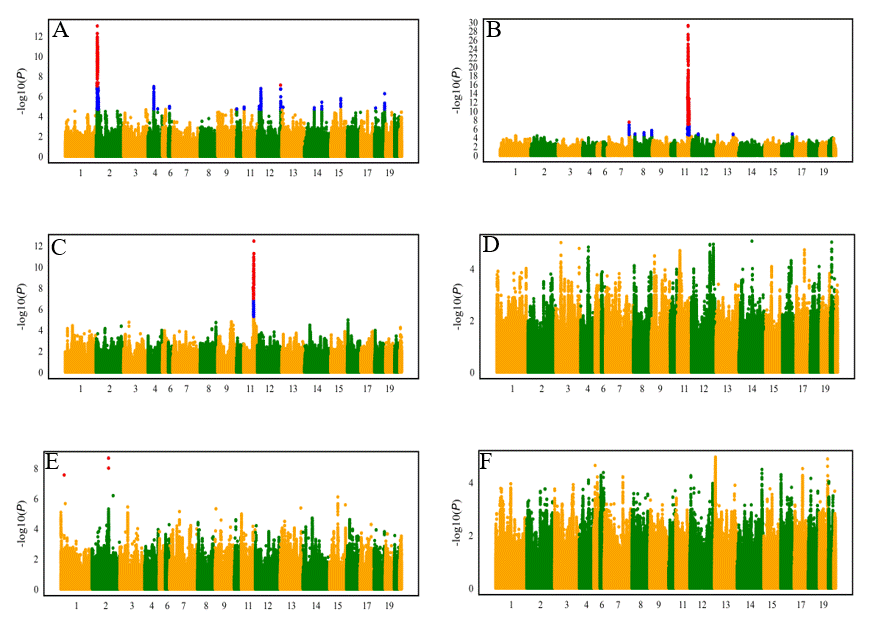

Supplement: Supplementary file 1 — Additional file 1: Figure S1. Venn diagram illustrating the number of the variants called by BaseVar, GATK and Bcftools. Figure S2. CLR and Pi analyses in the Angora rabbit population. Figure S3. Q-Q plots for the Angora rabbits. Figure S4. Manhattan plots for the Angora rabbits. [file 12711_2022_766_MOESM1_ESM.docx]
